# Abnormal composition and function of high‐density lipoproteins in atopic dermatitis patients

**DOI:** 10.1111/all.13620

**Published:** 2018-10-30

**Authors:** Markus Trieb, Peter Wolf, Eva Knuplez, Wolfgang Weger, Christian Schuster, Miriam Peinhaupt, Michael Holzer, Athina Trakaki, Thomas Eichmann, Achim Lass, Christian Wadsack, Rufina Schuligoi, Akos Heinemann, Gunther Marsche

**Affiliations:** ^1^ Division of Pharmacology Otto Loewi Research Center Medical University of Graz Graz Austria; ^2^ BioTechMed‐Graz Graz Austria; ^3^ Department of Dermatology Medical University of Graz Graz Austria; ^4^ Center of Explorative Lipidomics Graz Austria; ^5^ Institute of Molecular Biosciences University of Graz Graz Austria; ^6^ Department of Obstetrics and Gynaecology Medical University of Graz Graz Austria

To the Editor,

High‐density lipoprotein (HDL) is conserved and present in most species, suggesting an important biological role from an evolutionary standpoint. HDL suppresses the activation of immune cells,^1^ including eosinophils.^2^ Despite the evidence indicating that HDL is an important modulator of the immune response, not much is known about the role of HDL in human atopic diseases. Moreover, there is no information available whether allergy alters HDL composition and function.

In the present study, we isolated HDL from atopic dermatitis (AD) patients and control subjects and used mass spectrometry and biochemical analysis as well as cell‐based assays for detailed compositional and functional characterization. Methods and clinical characteristics of study subjects can be found in this article′s Data S1.

Strikingly, we identified complex alterations in the composition of AD‐HDL when compared to control HDL (Figure 1). As expected, the most abundant HDL protein was apolipoprotein (apo) A‐I (Figure 1A). AD‐HDL was enriched in apoA‐II (+12%, *P* = 0.0484) and the acute‐phase protein serum amyloid A (SAA) (+114%, *P* = 0.0250) when compared to control HDL, suggesting low‐grade inflammation (Figure 1B,F). However, median C‐reactive protein levels of AD patients were in the normal range (Table S1), suggesting only minimal inflammation. Of particular interest, levels of AD‐HDL‐associated apoC‐III (−70%, *P* < 0.0001) and apoE (−39%, *P* = 0.0014) were markedly decreased (Figure 1D,E), which appears to be unique, and were not reported in other disease states including psoriasis, an inflammatory skin disease.^3^ In good agreement with our results, a previous study demonstrated that the proteomic blood signature of AD is largely different from psoriasis.^4^ HDL‐associated apoA‐I and apoC‐II contents were not altered in AD patients (Figure 1A,C).

**Figure 1:**
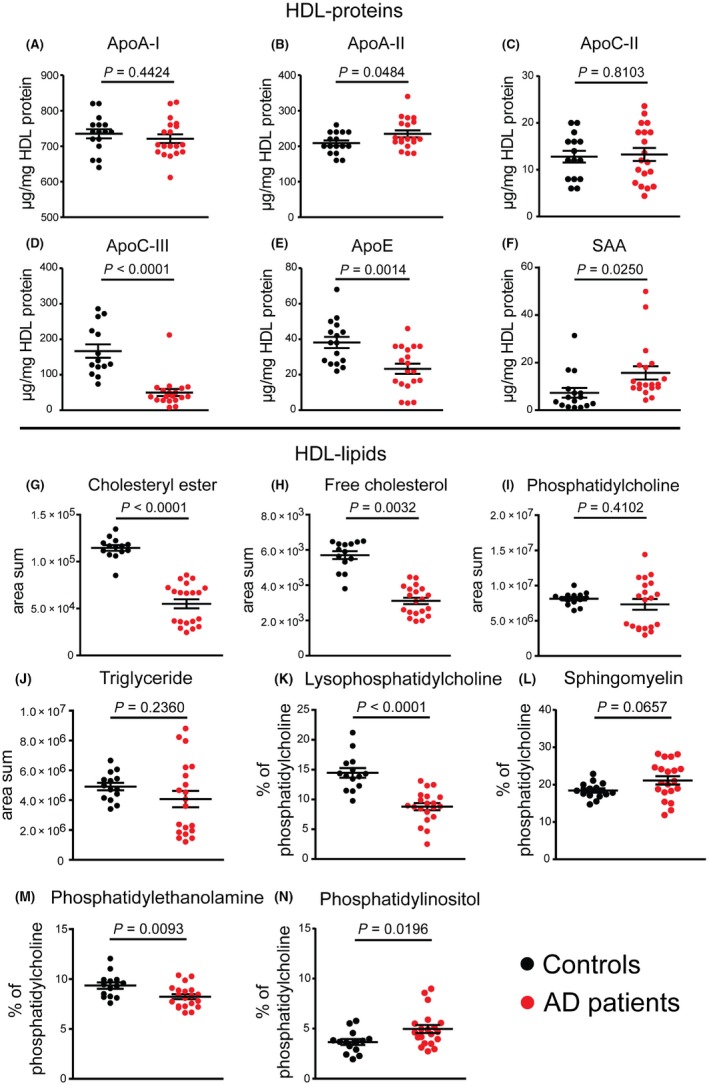
Protein and lipid pattern of high‐density lipoprotein (HDL). HDL was isolated from healthy subjects (control, n = 19) and patients with atopic dermatitis (n = 20) by ultracentrifugation and analyzed for its apolipoprotein (apo) A‐I (A), apoA‐II (B), apoC‐II (C), apoC‐III (D), and apoE (E) content by immunoturbidimetry. Serum amyloid A (SAA) content was measured using a commercially available ELISA (F). HDL contents of cholesteryl ester (G), free cholesterol (H), phosphatidylcholine (I), and triglycerides (J) were measured by mass spectrometry and are expressed as area sum. Relative amounts of lysophosphatidylcholine (K), sphingomyelin (L), phosphatidylethanolamine (M), and phosphatidylinositol (N) are expressed as percent to the total amount of PC. Levels of significance were calculated using Student's *t* test. Bars indicate mean and standard error of mean of the different test groups [Colour figure can be viewed at wileyonlinelibrary.com]

In addition, we observed striking low contents of HDL‐associated cholesteryl esters (−42%, *P* < 0.0001) (Figure 1G), free cholesterol (−45%, *P* = 0.0032) (Figure 1H), and lysophosphatidylcholine (LPC) (−39%, *P* < 0.0001) (Figure 1K). Moreover, AD‐HDL‐associated low abundant lipid species including phosphatidylethanolamine (−12%, *P* = 0.0093) (Figure 1M) and phosphatidylinositol differed (+36%, *P* = 0.0196) (Figure 1N), whereas contents of phosphatidylcholine, triglycerides, and sphingomyelin were not altered (Figure 1I,J,L). A detailed analysis of all analyzed lipid subspecies can be found in the Data S1 (Figure S1). Interestingly, this profoundly altered lipid composition of AD‐HDL is not seen in other disease states.^3^

Eosinophils are end‐stage effector cells inducing tissue damage in the inflammatory infiltrate within the dermis of AD patients.^5^ Isolated human eosinophils are suitable to conduct reproducible functional assays to test effector responses to HDL from AD patients and controls. We had to re‐isolate HDL from serum of AD patients and controls, given that storage damages HDL structure within 2 weeks.^6^ Sufficient serum to re‐isolate HDL was available from 16 patients and 8 controls. We pretreated eosinophils with HDL preparations, followed by stimulation with eotaxin‐2/CCL24 and monitored morphological changes (by flow cytometry) or performed chemotaxis assays using transwell plates. Strikingly, in contrast to HDL of controls, the majority of AD‐HDL samples promoted agonist induced chemotaxis (Figure 2A‐C) and did not suppress agonist induced shape change (Figure 2G‐I). A representative dot plot of eosinophil shape change is shown in Figure S2. An important HDL‐associated anti‐inflammatory enzyme is paraoxonase.^7^ Paraoxonase activity of most AD‐HDL samples was decreased when compared to control HDL (Figure 2N). Interestingly, we observed no change in the capacity of AD‐HDL to mobilize cholesterol from cells when compared to control HDL (Figure 2O).

**Figure 2:**
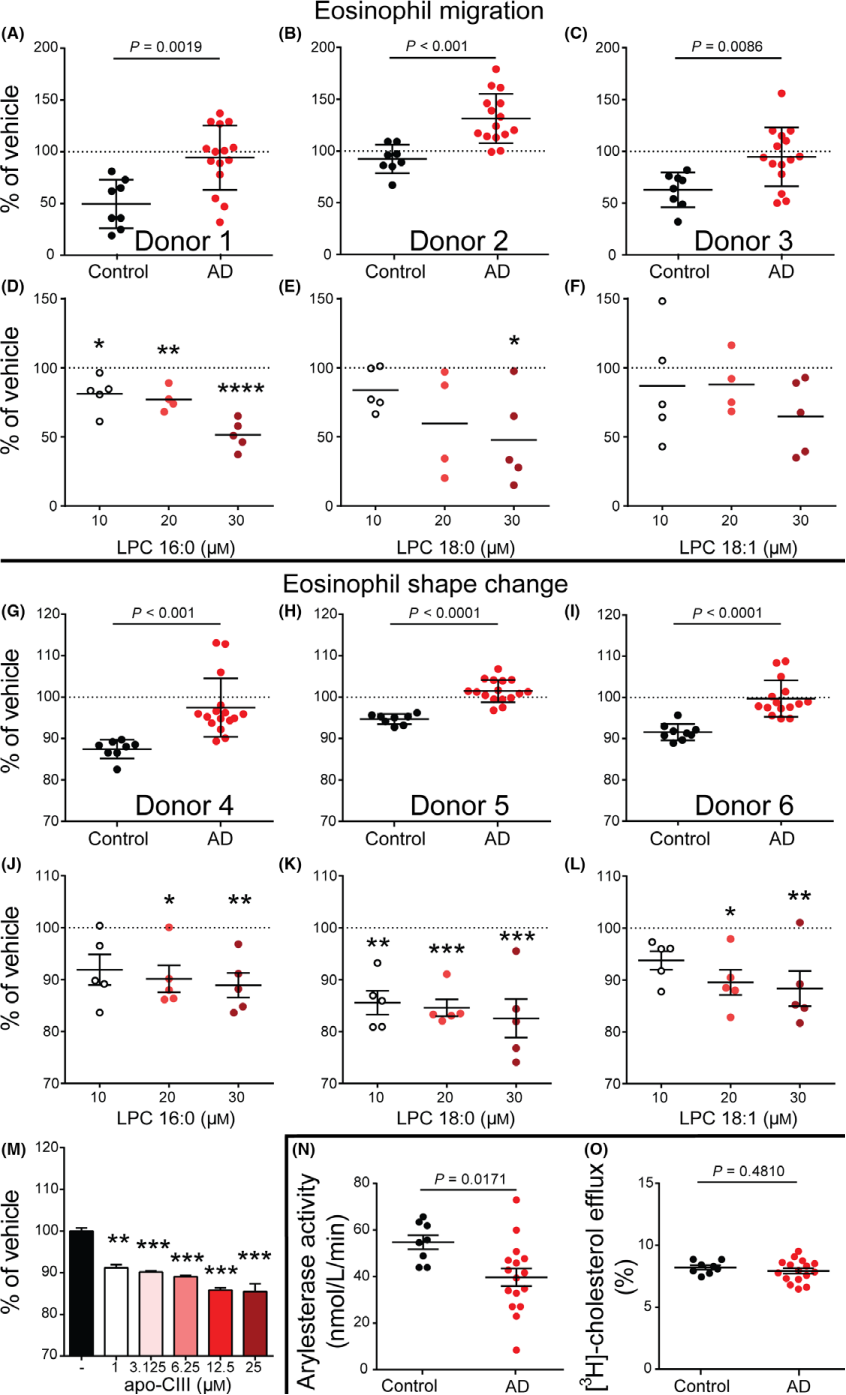
Metrics of high‐density lipoprotein (HDL) function. Isolated eosinophils of healthy, non‐atopic volunteers were pretreated with HDL (50 μg/mL) of control subjects (n = 8) and atopic dermatitis (AD) patients (n = 16) (**A‐C**) or indicated concentrations of lysophosphatidylcholine (LPC) 16:0 (**D**), LPC 18:0 (**E**) or LPC 18:1 (**F**). Samples were placed into transwell plate inserts with 5 μm pore size and allowed to migrate toward eotaxin‐2/CCL24 (30 nM) into the bottom well for 1 h at 37°C. Bars indicate mean and SEM of duplicate measurements of the different test groups (**A‐C**). Data are shown as mean and SEM from 5 individual donors (**D‐F**). Isolated eosinophils of healthy, non‐atopic volunteers were pretreated with HDL (50 μg/mL) of control subjects (n = 8) or AD patients (n = 16) (**G‐I**), indicated concentrations of LPC 16:0 (**J**), LPC 18:0 (**K**), LPC 18:1 (**L**) or indicated concentrations of apolipoprotein (apo)‐CIII (**M**) for 30 min and subsequently stimulated with eotaxin‐2/CCL24 (10 nM) for 4 mins at 37°C. Shape change was analyzed by flow cytometry as increase of forward scatter. Bars indicate mean and SEM of duplicate measurements of the different test groups (**G‐I**). Data are shown as mean and SEM of 5 individual donors (**J‐L**). Values shown as mean and SEM are representative of two independent experiments measured in duplicates (**M**). Responses were expressed as percent of vehicle (eotaxin‐2/CCL24 in the absence of HDL, apoC‐III or LPC) treated cells (**A‐M**). Arylesterase activity of HDL‐associated paraoxonase 1 of control subjects (n = 8) and AD patients (n = 16) was measured using phenylacetate as substrate (**N**). [^3^H]‐cholesterol‐labeled J774 macrophages were incubated with HDL (50 μg/mL) of control subjects (n = 8) and AD patients (n = 16) for 4 h (**O**). Cholesterol efflux is expressed as radioactivity in the supernatant relative to total radioactivity (in supernatant and cells). Values shown represent means of two individual experiments measured in duplicates (**N, O**). Bars indicate mean and SEM of the different test groups. Levels of significance were calculated using Student's t‐test or one‐way ANOVA and Dunnett post‐hoc test. *P < 0.05, **P < 0.01, ***P < 0.001, ****P < 0.0001 [Colour figure can be viewed at wileyonlinelibrary.com]

Prompted by the profound alterations in AD‐HDL composition and function, we performed a detailed correlation analysis to determine which proteins and lipids in AD‐HDL are associated with a loss of function. We observed multiple and complex associations (Table S2). Interestingly, the HDL‐triglyceride content correlated with the inhibitory activity of HDL toward agonist‐induced eosinophil shape change (*P* = 0.039) and migration (*P* = 0.008). HDL‐associated phospholipids and cholesteryl ester showed similar but weaker associations. SAA, an HDL‐associated pro‐inflammatory acute‐phase protein,^3^ correlated inversely with the ability to suppress agonist‐induced shape change of eosinophils (*P* = 0.001). However, agonist‐induced migration of eosinophils was not associated with SAA, suggesting that shape change and migration are affected by different components carried by HDL particles.

Other altered apolipoprotein contents in AD‐HDL could play a role in allergic diseases, given that apoE is an endogenous negative regulator of house dust mite–induced asthma in mice^8^ and apoC‐III affects catabolism of HDL.^3^ In line with that notion, we observed a strong correlation between the apoC‐III content of HDL and the HDL‐cholesterol content (HDL‐apoC‐III vs HDL‐cholesteryl ester: *r* = 0.541, *P* = 0.003; HDL‐apoC‐III vs HDL‐free cholesterol: *r* = 0.612, *P* < 0.001).

Moreover, AD‐HDL showed reduced levels of LPC (Figure 1K), especially LPC 16:0 (Figure S1, Data S1). We could recently demonstrate that HDL enriched with LPC 16:0 potently suppresses neutrophil effector responses.^9^ In agreement with our previous result, we observed that LPC 16:0 and LPC 18:0 dose‐dependently suppressed eosinophil shape change and migration (Figure 2D,E,J,K), while LPC 18:1 was able to reduce eosinophil shape change but had no inhibitory effect on eosinophil migration (Figure 2F,L). ApoC‐III levels were markedly decreased in AD‐HDL (Fig. 1D). Importantly, addition of recombinant apoC‐III reduced shape change of eosinophils (Fig. 2M), suggesting that the low contents of LPC and apoC‐III in AD‐HDL contribute to functional impairment. Further studies are needed to test whether other HDL‐associated proteins and lipids also affect eosinophil function.

Of note, we did not observe an impact of current treatment on HDL composition (Table S3) in our study.

We acknowledge limitations to this study. Due to the laborious experiments and analyses, we had to keep the patient number rather small. Therefore, additional studies with larger cohorts are required to confirm our results.

In conclusion, we show that AD is associated with profound alterations in HDL composition linked to the formation of dysfunctional HDL. Our results suggest a novel link between HDL dysfunction and allergy and may lead to new diagnostic and therapeutic approaches.

## CONFLICTS OF INTEREST

The authors declare that they have no conflicts of interest.
