# Supplementary material for: Abnormal composition and function of high‐density lipoproteins in atopic dermatitis patients
Source: Allergy. 2018 Oct 30;74(2):398–402. doi: 10.1111/all.13620 (PMC6491993; doi:10.1111/all.13620)
Supplement: Supplementary file 1 [file ALL-74-398-s001.docx]

**Repository:** **Abnormal Composition and Function of High Density Lipoproteins in Atopic Dermatitis Patients Trieb et al. 2018**

**Methods and extended results**

**Ethical approvals**

All procedures involving human subjects were approved by the Institutional Review Board of the Medical University of Graz and carried out in accordance with the Code of Ethics of the World Medical Association (Declaration of Helsinki). Informed consent was obtained from all subjects.

**Study subjects**

The clinical and laboratory characteristics of 20 study subjects with AD and 19 age- and sex-matched controls are given in Table E1. At study enrollment with consecutive recruitment (between March and June 2013), the majority of patients (n = 16) had long-lasting and widespread AD with affection of more than 10% of body surface area, high SCORAD values and very high total (> 1000 IU/ml) immunoglobulin E levels (n=13). Serum samples were stored at -70^o^C until analysis. The primary treatment modalities of the patients with AD included UVB-311nm phototherapy (n=5), antihistamines (n=2), oral steroids (n=1) and cyclosporin (n=3). Additional topical treatment with steroids (n=7), tacrolimus (n=2) and pimecrolimus (n=1) was given. The patients had decreased total and LDL-cholesterol levels, being in line with previous studies in German adults and Finnish children ^(1, 2)^. There was no relation of levels of total cholesterol, LDL-cholesterol and triglycerides to current treatment and none of the patients was taking lipid-lowering medication, such as statins.

**Determination of serum lipid composition**

Levels of total cholesterol and triglycerides (Diasys, Holzheim, Germany) were measured enzymatically. Low-density lipoprotein (LDL) cholesterol was calculated according to the Friedewald equation using HDL cholesterol values measured in the supernatant of the phosphotungstic acid/MgCl_2_ precipitation.

**Isolation of HDL**

Serum density was adjusted with potassium bromide (Sigma, Vienna, Austria) to 1.24 g/ml, and a two-step density gradient was generated in centrifuge tubes (16 × 76 mm, Beckman) by layering the density-adjusted serum underneath a KBr density solution (1.063 g/ml) as described ^(3)^. Tubes were sealed and centrifuged at 65,000 rpm for 6 h in a 90Ti fixed angle rotor (Beckman Instruments, Krefeld, Germany). After centrifugation, the HDL-containing band was collected, desalted via PD10 columns (GE Healthcare, Vienna, Austria) and immediately used for experiments or stored at -70°C in the presence of 10% glycerol as described ^(4)^.

**Biochemical quantification of HDL-associated proteins**

ApoA-I, apoA-II, apoC-II, apoC-III and apoE (Greiner, Flacht, Germany) were determined by immunoturbidimetry as described ^(5)^. All lipoprotein analyses were performed on an Olympus AU640 analyzer (Olympus Diagnostika, Hamburg, Germany).

SAA was determined by using an enzyme-linked immunosorbent assays (Life Technologies, Vienna, Austria) as described ^(6)^.

**Mass spectrometry analysis of HDL lipid composition**

Total lipids of HDL preparations (300 µg protein) were extracted twice according to Folch et al. ^(7)^ using chloroform/methanol/water (2/1/0.6, v/v/v) containing 500 pmol butylated hydroxytoluene, 1% acetic acid, and 100 pmol of internal standards (17:0-17:0 phosphatidylcholine, 17:0-17:0-17:0 triglyceride, 17:0 lysophosphatidylcholine (LPC), Avanti Polar Lipids, Alabama, AL) per sample. Extraction was performed under constant shaking for 60 min at room temperature. After centrifugation at 1,000 x g for 15 min at room temperature the lower organic phase was collected. 2.5 mL chloroform were added to the remaining aqueous phase and the second extraction was performed as described above. Combined organic phases of the double-extraction were dried under a stream of nitrogen and resolved in 450 µl 2-propanol/chloroform/methanol (7/2/1, v/v/v) for UPLC-qTOF analysis. Chromatographic separation was performed as described ^(8)^ with modifications, using an AQUITY-UPLC system (Waters Corporation), equipped with a HSS T3 column (2.1x100 mm, 1.8 µm; Waters Corporation). A SYNAPT™G1 qTOF HD mass spectrometer (Waters Corporation) equipped with an ESI source was used for detection. Data acquisition was done by the MassLynx 4.1 software (Waters Corporation) and lipid classes were analyzed with the “Lipid Data Analyzer 1.6.2” software ^(9)^. Data were normalized for recovery and extraction- and ionization efficacy using internal standards.

**Preparation of human eosinophils**

Blood was taken from healthy, non-atopic volunteers not taking any medication, according to a protocol approved by the Institutional Review Board of the Medical University of Graz.

Human peripheral blood eosinophils were isolated immediately from serum as described before ^(10)^ and were used for experiments within 4 hours.

**Shape change assay**

Isolated eosinophils (25,000 maintained in PBS with Ca^2+^ and Mg^2+^, HEPES 10 mmol/L, glucose 10 mmol/L and bovine serum albumin 0.1%, pH 7.4) were pretreated with HDL (50 µg/mL) for 30 minutes and subsequently stimulated with eotaxin-2/CCL24 (10 nM) (Immunotools, Friesoythe, Germany) for 4 minutes at 37°C. Afterwards, samples were fixed with BD CellFIX solution (BD Biosciences, Franklin Lakes, NJ, USA) and analyzed by flow cytometry. Shape change was estimated as increase of forward scatter ^(11)^.

**Chemotaxis assay**

Isolated eosinophils (50,000 maintained in PBS with Ca^2+^ and Mg^2+^, HEPES 10 mmol/L, glucose 10 mmol/L and bovine serum albumin 0.1%, pH 7.4) were pretreated with HDL (50 µg/mL) for 30 minutes. Then, cells were placed into Transwell inserts with 5 µm pore size filters (Corning, Acton, MA, USA). Cells were allowed to migrate toward eotaxin-2/CCL24 (30 nM) (Immunotools, Friesoythe, Germany) into the bottom well for 1 hour at 37°C. Afterwards, migrated cells were fixed with BD CellFIX solution (BD Biosciences, Franklin Lakes, NJ, USA) and counted by flow cytometry.

**Arylesterase activity assay**

Ca^2+^-dependent arylesterase activity was determined with a photometric assay using phenylacetate as substrate as described ^(12)^.

**HDL cholesterol efflux capability**

Cholesterol efflux capacity was assessed using a validated assay ^(4)^.

**Statistical analysis**

Differences between two groups were analyzed with the Student’s t-test (unpaired, two-tailed). Comparison of normal distributed groups was performed with One-Way ANOVA and Bonferroni post-hoc test. Correlations were determined using the Pearson product-moment estimates. Group differences were considered statistically significant for **p* < 0.05, ***p* < 0.01, ****p* < 0.001 and **** *p* < 0.0001. Statistical analyses were performed using GraphPad Prism (Version 4.0, GraphPad Software) or SPSS Statistics Version 22.


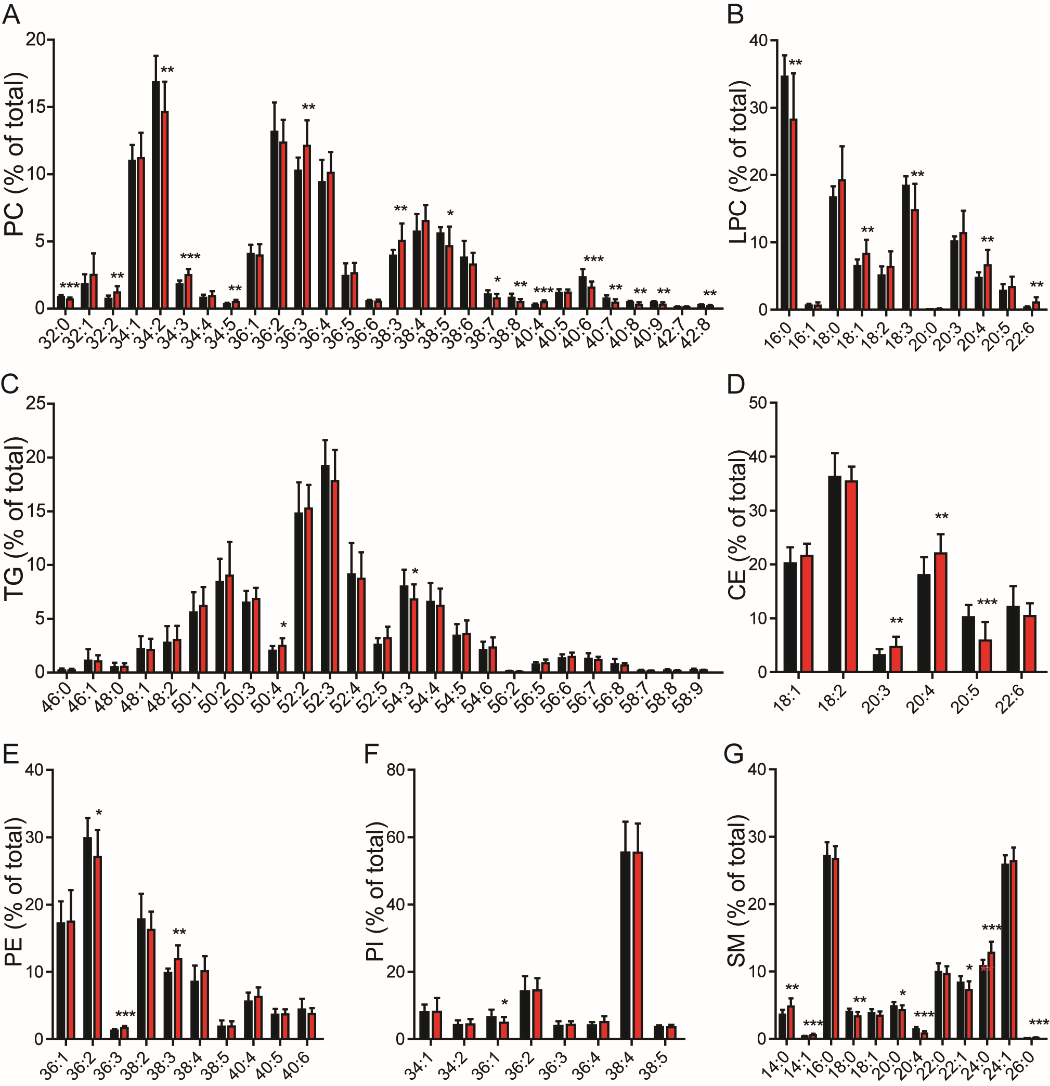
**Figures**

**Figure E1:** **Detailed analysis of lipid species of HDL of healthy controls (black bars) and patients with atopic dermatitis (red bars).** Analysis showing the prevalence of chain length variants for phosphatidylcholine (PC), triglycerides (TG), lysophosphatidylcholine (LPC), cholesteryl ester (CE), phosphatidylethanolamine (PE), phosphatidylinositol (PI) and sphingomyelin (SM) (A – G). **p* < 0.05, ***p* < 0.01, ****p* < 0.001.


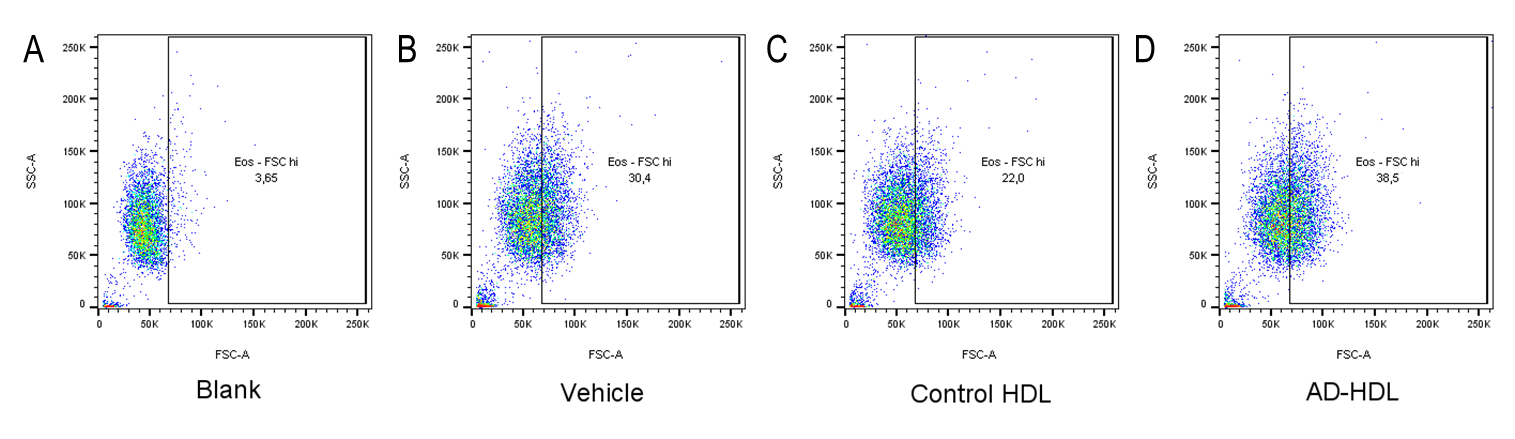


**Figure E2:** **Shape change of isolated eosinophils from a healthy, non-atopic volunteer.** Forward scatter (FSC) versus sideward scatter (SSC) dot plots of (A) unstimulated eosinophils or (B-D) eosinophils stimulated with eotaxin-2/CCL24. (C) Eosinophils pretreated with control HDL. (D) Eosinophils pretreated with HDL of a patients with atopic dermatitis (AD-HDL). Representative values are shown.

| **Tables**  **Table E1. Clinical characteristics of the study subjects** | | | |
| --- | --- | --- | --- |
|  | **Controls** | **Atopic dermatitis** | ***p*** |
| **n** | 19 | 20 |  |
| **Age** (y) | 31 (26-38) | 33 (19-50) | 0.2598 |
| **Male/female** | 7 / 12 | 7 / 13 | 0.9080 |
| **Total cholesterol** (mg/dL) | 174 (151-199) | 144 (137-179) | 0.0087 |
| **Triglycerides** (mg/dL) | 78 (56-123) | 61 (40-95) | 0.0876 |
| **HDL-cholesterol** (mg/dL) | 51 (44-70) | 55 (45-74) | 0.4279 |
| **LDL-cholesterol** (mg/dL) | 107 (78-121) | 73 (62-109) | 0.0099 |
| **Creatinin** (mg/dL) | n.d. | 1.4 (1-7) | - |
| **SCORAD** | n.d. | 48 (39-61) | - |
| **CRP** (mg/L) | n.d. | 1.3 (0.6-6.9) | - |
| **IgE** (U/mL) | n.d. | 3453 (198-5000) | - |

Values are given as medians with the interquartile range. n.d.: not determined; SCORAD, scoring atopic dermatitis; CRP, C-reactive protein; IgE, immunoglobulin E.

| **Table E2. Correlation of HDL composition with HDL function of AD patients (n=16)** | | | | | | | |
| --- | --- | --- | --- | --- | --- | --- | --- |
|  | **Shape change**  (mean value) | | **Chemotaxis**  (mean value) | | **Paraoxonase activity**  (mean value) | |  |
|  | **r** | ***p*** | **r** | ***p*** | **r** | ***p*** |  |
| **HDL-ApoA-I** (µg/mg protein) | -0.048 | 0.860 | -0.291 | 0.292 | 0.222 | 0.408 |  |
| **HDL-ApoA-II** (µg/mg protein) | -0.128 | 0.637 | -0.282 | 0.308 | -0.400 | 0.125 |  |
| **HDL-ApoC-II** (µg/mg protein) | 0.374 | 0.154 | 0.331 | 0.229 | 0.013 | 0.963 |  |
| **HDL-ApoC-III** (µg/mg protein) | 0.454 | 0.077 | -0.050 | 0.858 | -0.231 | 0.389 |  |
| **HDL-ApoE** (µg/mg protein) | 0.458 | 0.074 | 0.289 | 0.296 | 0.210 | 0.435 |  |
| **HDL-SAA** (µg/mg protein) | 0.731 | 0.001^#^ | 0.238 | 0.394 | 0.113 | 0.677 |  |
| **HDL-PC** (area sum) | -0.509 | 0.044^#^ | -0.584 | 0.022^#^ | -0.046 | 0.865 |  |
| **HDL-TG** (area sum) | -0.521 | 0.039^#^ | -0.654 | 0.008^#^ | -0.174 | 0.520 |  |
| **HDL-CE** (area sum) | -0.545 | 0.029^#^ | -0.414 | 0.125 | -0.201 | 0.456 |  |
| **HDL-FC** (area sum) | -0.457 | 0.075 | -0.450 | 0.092 | 0.019 | 0.943 |  |
| **HDL-LPC** (% of PC) | 0.095 | 0.728 | -0.066 | 0.814 | -0.003 | 0.992 |  |
| **HDL-SM** (% of PC) | -0.601 | 0.014^#^ | -0.400 | 0.140 | -0.036 | 0.895 |  |
| **HDL-PE** (% of PC) | -0.282 | 0.290 | 0.079 | 0.779 | 0.409 | 0.116 |  |
| **HDL-PI** (% of PC) | -0.267 | 0.318 | -0.094 | 0.740 | 0.084 | 0.757 |  |

Shape change and chemotaxis of eosinophils were each analyzed in 3 individual donors measured in duplicates. Paraoxonase was measured in two individual experiments measured in duplicates. Apo, apolipoprotein; SAA, serum amyloid A; PC, phosphatidylcholine; TG, triglyceride; CE, cholesteryl ester; FC, free cholesterol; LPC, lysophosphatidylcholine; SM, sphingomyelin; PE, phosphatidylethanolamine; PI, phosphatidylinositol. ^#^ significant correlation.

| **Table E3. Impact of treatment on HDL composition** | | | |
| --- | --- | --- | --- |
|  | **Current treatment** | |  |
|  | **no (n=13)** | **yes (n=7)** | ***p*** |
| **HDL-SAA** (µg/mg protein) | 11.5 (7.9-19.5) | 10.8 (9.7-17.9) | 0.56 |
| **HDL-PC** (area sum) | 8.2×10^6^ (4.1×10^6^-1.1×10^7^) | 8.0×10^6^ (3.9×10^6^-9.0×10^6^) | 0.79 |
| **HDL-TG** (area sum) | 4.1×10^6^ (1.8×10^6^-5.6×10^6^) | 3.6×10^6^ (2.0×10^6^-6.3×10^6^) | 0.85 |
| **HDL-CE** (area sum) | 6.7×10^4^ (3.3×10^4^-7.2×10^4^) | 6.7×10^4^ (3.5×10^4^-7.7×10^4^) | 0.90 |
| **HDL-SM** (% of PC) | 21.0 (18.1-24.4) | 20.1 (15.4-28.1) | 0.91 |

Values are given as medians with the interquartile range. SAA, serum amyloid A; PC, phosphatidylcholine; TG, triglyceride; CE, cholesteryl ester; SM, sphingomyelin. The primary treatment modalities of patients with atopic dermatitis included UVB 311nm phototherapy (n=2), oral cyclosporine (n=1) and topical steroids (n=4). One patient was additionally treated with topical tacrolimus.

**References**

1. Schafer T, Ruhdorfer S, Weigl L, Wessner D, Heinrich J, Doring A*, et al*. Intake of unsaturated fatty acids and HDL cholesterol levels are associated with manifestations of atopy in adults. Clin Exp Allergy. 2003 Oct;33(10):1360-7.

2. Pesonen M, Ranki A, Siimes MA, Kallio MJ. Serum cholesterol level in infancy is inversely associated with subsequent allergy in children and adolescents. A 20-year follow-up study. Clin Exp Allergy. 2008 Jan;38(1):178-84.

3. Holzer M, Birner-Gruenberger R, Stojakovic T, El-Gamal D, Binder V, Wadsack C*, et al*. Uremia alters HDL composition and function. J Am Soc Nephrol. 2011 Sep;22(9):1631-41.

4. Holzer M, Kern S, Trieb M, Trakaki A, Marsche G. HDL structure and function is profoundly affected when stored frozen in the absence of cryoprotectants. J Lipid Res. 2017 Sep 11.

5. Holzer M, Trieb M, Konya V, Wadsack C, Heinemann A, Marsche G. Aging affects high-density lipoprotein composition and function. Biochim Biophys Acta. 2013 Sep;1831(9):1442-8.

6. Pertl L, Kern S, Weger M, Hausberger S, Trieb M, Gasser-Steiner V*, et al*. High-density lipoprotein function in exudative age-related macular degeneration. PLoS One. 2016 May 12;11(5):e0154397.

7. FOLCH J, LEES M, SLOANE STANLEY GH. A simple method for the isolation and purification of total lipides from animal tissues. J Biol Chem. 1957 May;226(1):497-509.

8. Knittelfelder OL, Weberhofer BP, Eichmann TO, Kohlwein SD, Rechberger GN. A versatile ultra-high performance LC-MS method for lipid profiling. J Chromatogr B Analyt Technol Biomed Life Sci. 2014 Mar 1;951-952:119-28.

9. Hartler J, Trotzmuller M, Chitraju C, Spener F, Kofeler HC, Thallinger GG. Lipid data analyzer: Unattended identification and quantitation of lipids in LC-MS data. Bioinformatics. 2011 Feb 15;27(4):572-7.

10. Konya V, Blattermann S, Jandl K, Platzer W, Ottersbach PA, Marsche G*, et al*. A biased non-galphai OXE-R antagonist demonstrates that galphai protein subunit is not directly involved in neutrophil, eosinophil, and monocyte activation by 5-oxo-ETE. J Immunol. 2014 May 15;192(10):4774-82.

11. Frei RB, Luschnig P, Parzmair GP, Peinhaupt M, Schranz S, Fauland A*, et al*. Cannabinoid receptor 2 augments eosinophil responsiveness and aggravates allergen-induced pulmonary inflammation in mice. Allergy. 2016 Jul;71(7):944-56.

12. Holzer M, Wolf P, Inzinger M, Trieb M, Curcic S, Pasterk L*, et al*. Anti-psoriatic therapy recovers high-density lipoprotein composition and function. J Invest Dermatol. 2014 Mar;134(3):635-42.
